# Influence of Storage Conditions on Physical Properties of Freeze-Dried Vegetable Snacks Packed in Pork Gelatin Films

**DOI:** 10.3390/molecules31040747

**Published:** 2026-02-22

**Authors:** Agnieszka Ciurzyńska, Magdalena Karwacka, Karolina Szulc, Klaudia Wieczorek, Monika Janowicz, Sabina Galus

**Affiliations:** Department of Food Engineering, Institute of Food Sciences, Warsaw University of Life Sciences-SGGW, Nowoursynowska Str., 159c, 02-776 Warsaw, Poland; magdalena_karwacka@sggw.edu.pl (M.K.); karolina_szulc@sggw.edu.pl (K.S.); s204174@sggw.edu.pl (K.W.); monika_janowicz@sggw.edu.pl (M.J.); sabina_galus@sggw.edu.pl (S.G.)

**Keywords:** edible film, storage, freeze-dried snacks, color, structure

## Abstract

The aim of this study was to investigate the changes in selected physical properties of freeze-dried vegetable snacks packed in edible films based on pork gelatin of different concentrations (8 and 12%), during storage at temperatures of 4 °C and 20 °C for periods of 3 and 6 months. The scope of this work includes the preparation of freeze-dried carrot snacks, obtaining edible films, packaging the snacks, and testing selected physical properties. The results show that storage time and temperature significantly affected the quality of the freeze-dried snacks. Water activity increased from an initial value of approximately 0.12 in the control samples to values ranging between 0.27 and 0.60 after storage, depending on gelatin concentration, temperature, and storage duration. The lowest water activity values (≈0.27–0.28) were observed for samples stored at 20 °C for 3 months, regardless of gelatin concentration, whereas storage for 6 months resulted in water activity values close to 0.5–0.6. Dry matter content decreased from about 97% in the control samples to values ranging from approximately 73.6% to 87.0% for samples coated with 8% gelatin and from 78.5% to 86.7% for samples coated with 12% gelatin, with greater reductions observed at longer storage times and lower storage temperature. Mechanical analysis indicated a strengthening of product structure after 3 months of storage, followed by a marked reduction in compression force—almost tenfold—after 6 months, indicating structural weakening. Color saturation (C) increased after 3 months of storage (values around 40–42), but significantly decreased after 6 months, reaching values as low as approximately 13–24, particularly at 20 °C. Porosity remained high throughout storage, generally in the range of 94–95%, although microscopic analysis revealed progressive pore collapse after 6 months of storage. Overall, a storage temperature of 20 °C and a storage time of 3 months were identified as the most favorable conditions for freeze-dried carrot snacks packed in edible films with both 8% and 12% gelatin, ensuring lower water activity, higher dry matter content, and better structural stability.

## 1. Introduction

Food packaging has a significant effect on preserving the quality of fresh and processed foods. The primary function of packaging is to protect food quality and prevent spoilage and contamination from microorganisms from its production to consumption [1]. Food packaging should be convenient to use and easy to open, close, and store. It is also important that it is ecological, i.e., suitable for recycling or biodegradation. Research has been ongoing for years to create packaging with all of the above-mentioned features [2,3].

Films produced from natural and edible materials have emerged as a promising alternative to conventional packaging systems. These materials are fully edible and biodegradable and can be applied as coatings to foods of different shapes and sizes. Edible films are capable of protecting food products against moisture loss, microbial contamination, and external environmental factors at a level comparable to that of conventional plastic packaging [4]. Edible packaging is made of natural materials. Their composition makes them biodegradable and environmentally friendly because production reduces the use of fossil fuels and carbon dioxide emissions [5]. Currently, the composition of edible coatings is primarily based on natural compounds, including proteins, lipids, and polysaccharides [6]. The main function of edible coatings is the selective barrier to water vapor, oxygen, carbon dioxide, aromatic substances, and oils [7].

Protein or polysaccharide films are characterized by good mechanical properties. At low or medium relative humidity, they are a strong barrier to oxygen and a weak barrier to water vapor, which results from their hydrophilic nature [8]. Proteins are characterized by excellent mechanical properties and are flexible, colorless, and odorless [9]. Gelatin is a natural soluble protein, gelling or non-gelling, obtained by partial hydrolysis of collagen from bones, tendons, skin, cartilage and other animal tissues [10]. Dissimilar food products and storage conditions need specific packaging considerations [1]. Edible gelatin-based films can be a promising solution to the problem of packaging for freeze-dried snacks, which are popular due to their long shelf life and high quality, but their storage can lead to changes in physical properties due to the high hygroscopicity of the freeze-dried product [11,12]. Previous investigations of Ciurzyńska et al. [13] concerned the effect of storage conditions on selected physical properties of freeze-dried carrot snacks coated with varying concentrations of pork gelatin by immersing dried material in a film-forming solution. The differences in the coating method between applying a coating by immersing the freeze-dried product in a film-forming solution and wrapping the product in a pre-formed edible film affect the properties and quality of the product, especially in the surface layer. The coating layer applied by immersing the product becomes an integral part of the sample, while the edible film can be removed at any time, leaving the freeze-dried product as just that. Therefore, comparing the presented research results with those previously published on immersion coating will complement and expand the current knowledge on this topic. Determining the changes in physical properties during storage of freeze-dried products packaged in edible films appears to be an interesting research topic and will allow for a comprehensive understanding of the optimal packaging and storage conditions for freeze-dried products. To obtain a complete picture of the physical properties, it was decided to examine changes in the properties of freeze-dried products packaged in 8% and 12% concentration films, as it was uncertain whether the gelatin concentration would reveal a more pronounced effect on the strength of the edible packaging and the retention of the freeze-dried product’s properties during sample storage.

The aim of the present study was to investigate changes in the selected physical properties of freeze-dried vegetable snacks packed in gelatin-based edible films with different concentrations (8 and 12%) and stored at temperatures of 4 and 20 °C for periods of 3 and 6 months. The scope of this study included the preparation of freeze-dried carrot snacks, the production of two variants of pork gelatin edible films, the evaluation of selected physical properties after storage, and the analysis of the obtained results.

## 2. Results

### 2.1. Effect of Storage Conditions on the Water Activity and Dry Matter Content of Freeze-Dried Vegetable Snacks Packed in Edible Films

Based on the statistical analysis of the obtained results, it was found that storage of freeze-dried snacks coated with an edible film with a gelatin concentration of 8% (Table 1) led to a statistically significant increase in water activity compared to the control sample. Storage at 4 °C resulted in a significantly higher water activity of the freeze-dried samples, whereas storage at 20 °C proved to be more advantageous, as it yielded lower water activity values. Nevertheless, extending the storage period from 3 to 6 months caused a further increase in water activity for samples stored at 20 °C, indicating progressive moisture uptake over time. The control sample exhibited a dry matter content of approximately 97% (Table 1). Packaging freeze-dried snacks in gelatin-based edible films with an 8% gelatin concentration resulted in a statistically significant reduction in dry matter content, reaching 77.61% for samples stored at 4 °C for 3 months and 87.03% for samples stored at 20 °C for the same period.

Also, in the case of samples coated with a 12% gelatin film (Table 2), storage at 4 and 20 °C significantly increased the water activity of the freeze-dried samples, and extending the storage time from 3 to 6 months caused a further increase in the tested parameter to a value close to 0.5 and 0.6. For the dry matter content of samples packed in gelatin films with a gelatin content of 12% (Table 2), similar dependencies were obtained, as in the case of films with a gelatin content of 8% (Table 1). Regardless of the storage temperature, they were characterized by a decrease in the dry matter content compared to the control sample (approx. 97%). In samples stored for 3 months at a storage temperature of 4 °C, the dry matter content was 82.86%, while for the sample stored at 20 °C, it was 86.67%. Extending the storage time from 3 to 6 months decreased the dry matter content, but only for the storage temperature of 4 °C; differences were statistically significant.

### 2.2. Effect of Temperature and Storage Time on the Color of Freeze-Dried Vegetable Snacks Packed in Edible Films

**Figure 1:**
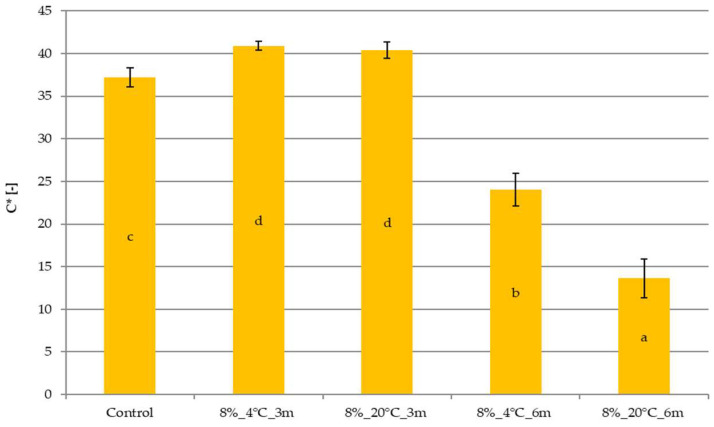
The effect of temperature and storage time on the color saturation coefficient of freeze-dried vegetable snacks packed in edible film based on 8% pork gelatin. The same letter (^a–d^) indicates no statistically significant differences (*p* < 0.05). Designation of samples—Table 4.

**Figure 2:**
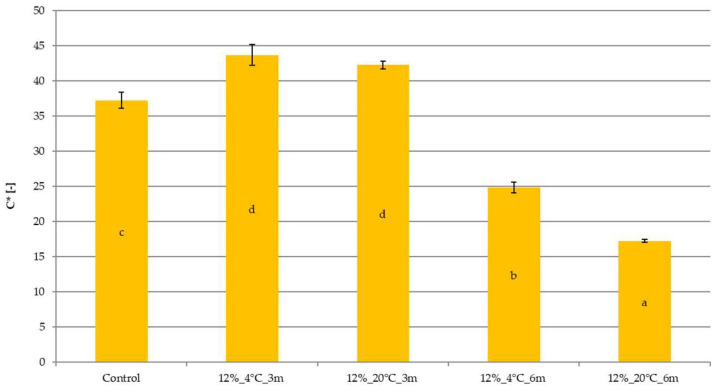
The effect of temperature and storage time on the color saturation coefficient of freeze-dried vegetable snacks packed in edible film based on 12% pork gelatin. The same letter (^a–d^) indicates no statistically significant differences (*p* < 0.05). Designation of samples—Table 4.

The results of the color saturatuion coefficient (C*) are presented in Figure 1 and Figure 2. The control sample was characterized by a color saturation coefficient of about 37 (Figure 1). Storage in films with an 8% gelatin concentration resulted in a statistically significant increase in this parameter to approximately 41, but only after 3 months. Extending the storage time from 3 to 6 months led to a decrease in the color saturation index, which reached the lowest values of 24.03 for sample 8%_4 °C_6m and 13.64 for sample 8%_20 °C_6m.

Similar color changes were obtained for samples stored in edible film with a gelatin concentration of 12% (Figure 2). The color saturation coefficient (C*) significantly increased after 3 months of storage, whereas after 6 months, it significantly decreased, reaching the lowest value for sample 12%_20°C_6m.

### 2.3. Influence of Storage Conditions on the Mechanical Properties of Freeze-Dried Vegetable Snacks Packed in Edible Films

**Figure 3:**
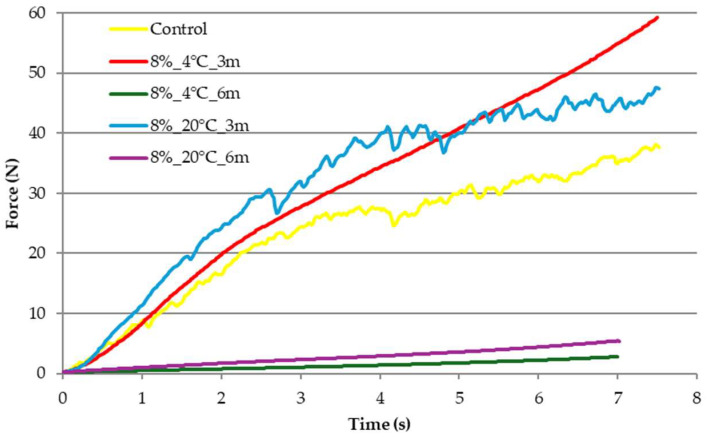
Effect of temperature and storage time on the mechanical properties of freeze-dried vegetable snacks packed in edible film based on 8% pork gelatin. Designation of samples—Table 4.

**Figure 4:**
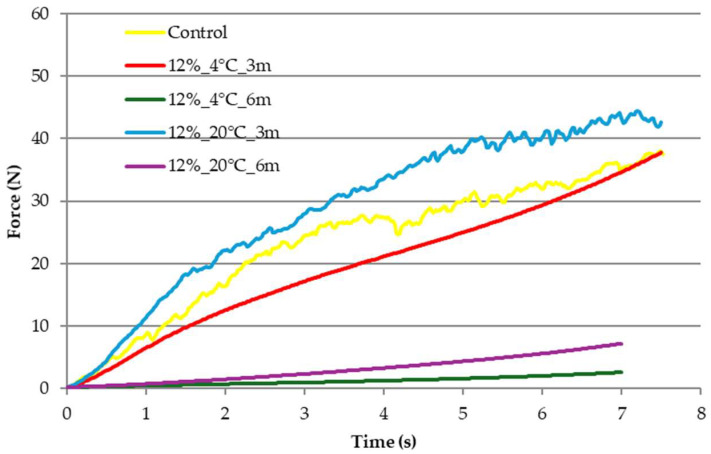
Effects of temperature and storage time on the mechanical properties of freeze-dried vegetable snacks packed in edible film based on 12% pork gelatin. Designation of samples—Table 4.

Figure 3 and Figure 4 show the curves of the deformation force of freeze-dried carrot snacks over time. Based on the obtained compression curves, it was shown that freeze-dried snacks packed in edible film with 8% gelatin concentration storage at temperatures of 4 and 20 °C for 3 months required the use of a greater compression force than the control sample (Figure 3). Based on the course of the curve, it was found that storing freeze-dried products at 4 °C for 3 months reduced the brittleness typically associated with freeze-dried products. On the other hand, the compression curve obtained for the sample stored at 20 °C for 3 months was similar to the control sample. Extending the storage time to 6 months for both temperatures resulted in a noticeable weakening of the freeze-dried product structure. Their deformation required almost 10 times lower compression force.

In the case of samples stored in edible film with a gelatin concentration of 12%, similar changes in mechanical properties were noted (Figure 4). Storage at a temperature of 20 °C for 3 months resulted in a strengthening of the structure of the dried products in relation to the control sample, and the compression curve is similar to the curve of the sample packed in edible film with a concentration of 8% (Figure 3) and stored at the same temperature, as well as to the control sample. After 3 months at 4 °C, the brittleness typical of freeze-dried products decreased, and the compression curve shows a smooth course while also achieving high mechanical resistance. Extending the storage time to 6 months for both temperatures resulted in a significant weakening of the structure. Their deformation also required an almost ten times lower compression force.

### 2.4. Effect of Storage Conditions on the Porosity of Freeze-Dried Vegetable Snacks Packed in Edible Films

**Figure 5:**
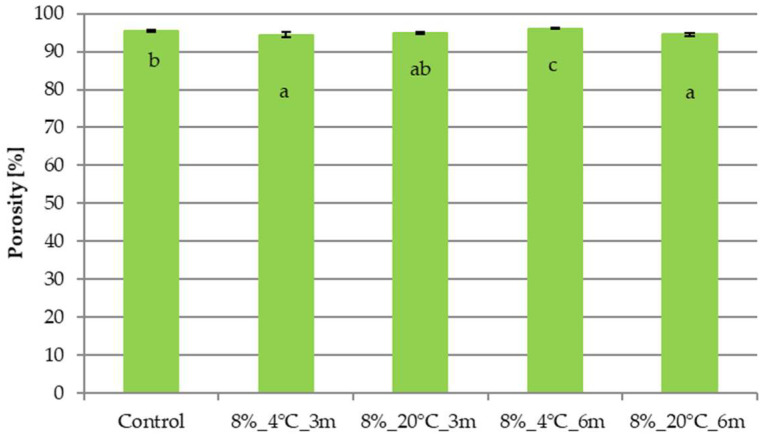
Effect of temperature and storage time on the porosity of freeze-dried vegetable snacks packed in edible film based on 8% pork gelatin. The same letter (^a–c^) indicates no statistically significant differences (*p* < 0.05). Designation of samples—Table 4.

Figure 5 presents the porosity results for samples stored in gelatin edible films with an 8% gelatin concentration. The porosity of the control sample was 95.42%. Storage of freeze-dried snacks packaged in gelatin edible films with an 8% gelatin concentration, in most cases, decreased the porosity of the freeze-dried product. Extending the storage time from 3 to 6 months resulted in a significant increase in the tested parameter only for the sample stored at 4 °C.

**Figure 6:**
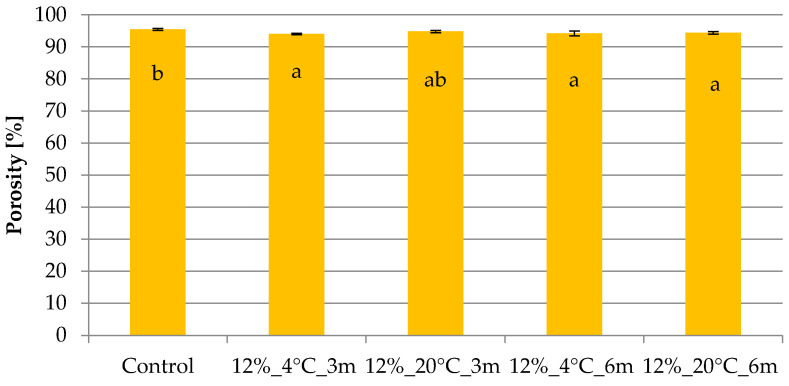
Effect of temperature and storage time on the porosity of freeze-dried vegetable snacks packed in edible film based on 12% pork gelatin. The same letter (^a,b^) indicates no statistically significant differences (*p* < 0.05). Designation of samples—Table 4.

Figure 6 presents the results of the porosity test for samples stored in gelatin edible films with a gelatin concentration of 12%. In the case of samples storage for 3 and 6 months, a significant decrease in porosity was demonstrated compared to the porosity of the control sample (95.42%), although it remained at a high level, ~94–95%. Extending the storage time to 6 months resulted in insignificant porosity changes.

### 2.5. Effect of Storage Conditions on the Structure of Freeze-Dried Vegetable Snacks Packed in Edible Films

**Figure 7:**
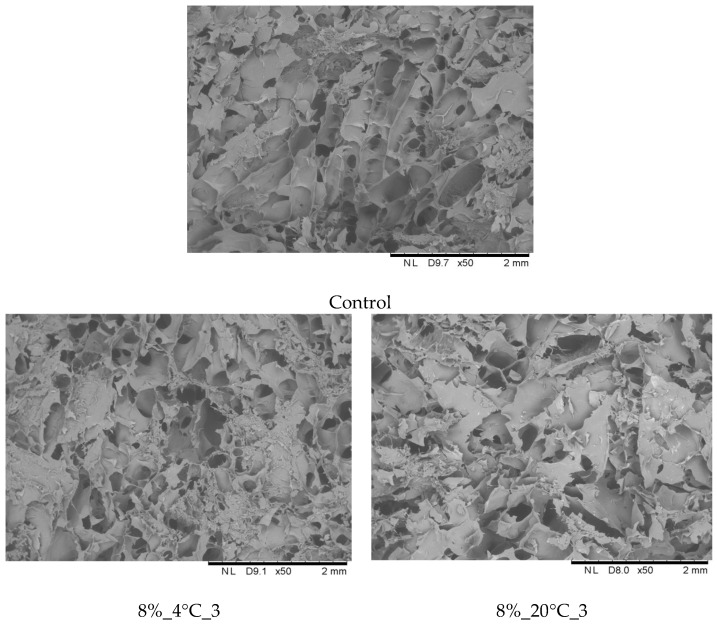

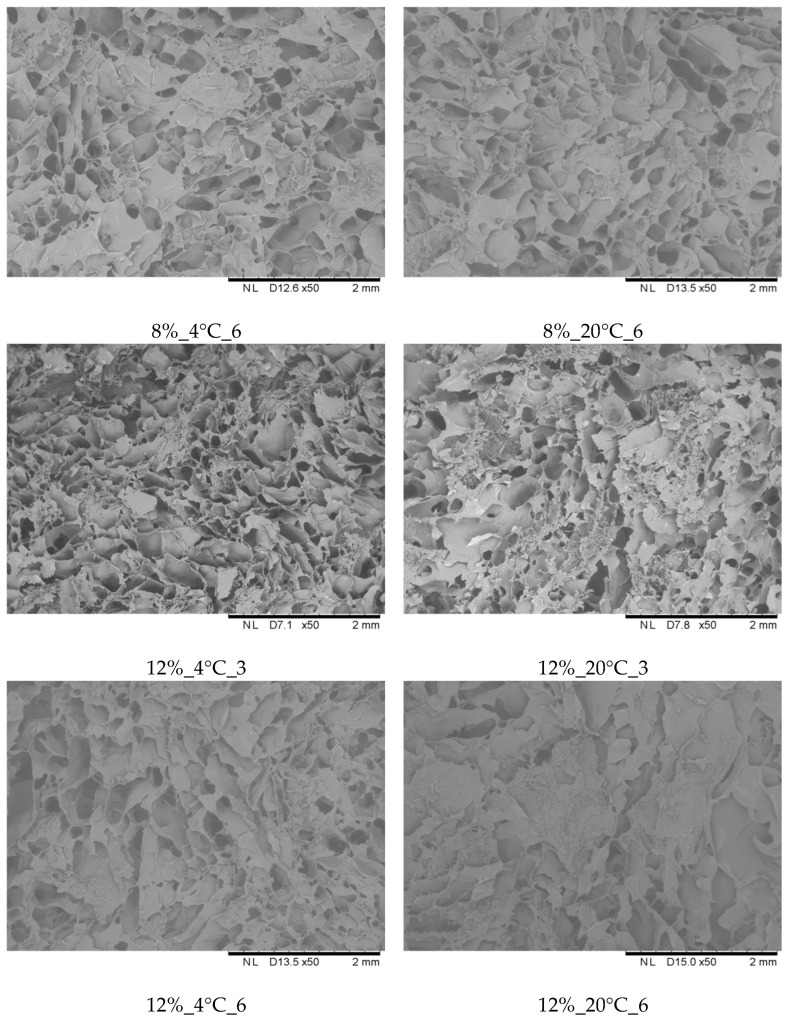
Effect of temperature and storage time on the internal structure of freeze-dried vegetable snacks packed in edible film based on 8 and 12% pork gelatin. Designation of samples—Table 4.

Figure 7 presents micrographs of the internal structure of freeze-dried snacks stored in 8% and 12% gelatin films for 3 and 6 months, compared with the control sample. Samples stored for 3 months retained high porosity, exhibiting internal structures similar to those of the control sample. In contrast, extending the storage time from 3 to 6 months at both 4 and 20 °C resulted in a pronounced structural collapse, characterized by a marked reduction in the number of open pores.

## 3. Discussion

Water activity is a critical parameter in processed foods, as it must be reduced to inhibit microbial growth and maintain product stability. It represents the availability of water for microbial metabolism, and an increase in this parameter directly favors microbial development [14]. The minimum water activity required for microbial growth is approximately 0.6; most bacteria grow optimally at values around 0.91, fungi at approximately 0.8, and yeasts at about 0.61 [15]. In the present study, the lowest water activity values for carrot snacks stored in edible films with different gelatin concentrations were observed after 3 months of storage at 20 °C (≈0.28). Extending the storage period to 6 months increased water activity to values close to 0.5, indicating progressive moisture uptake. This behavior can be explained by the findings of García et al. [16], who reported that glycerol-plasticized films exhibit higher gas and vapor permeability than starch-based films plasticized with sorbitol; however, based on the water activity values of the samples, it can be concluded that increasing the glycerol concentration, along with increasing the gelatin concentration in the edible films, caused slight changes in the tested parameter, and films have similar barrier properties. Similar trends were reported for samples coated by immersion in edible coatings, as described by Ciurzyńska et al. [13]. Ignaczak et al. [17] reported water activity values of approximately 0.294, which are comparable to those obtained for freeze-dried carrot snacks stored for 3 months in gelatin-based edible films containing 8% and 12% gelatin, confirming the effectiveness of edible films as packaging materials for low-moisture products. The higher water activity of samples storage at 4 °C may be related to the higher relative humidity at 4 °C (59.4%), and the product is more susceptible to moisture sorption from the environment compared to air humidity at 20 °C (35.3%) [13].

The control sample exhibited a dry matter content of 97.3%, which is consistent with the values reported by Ciurzyńska et al. [18] for freeze-dried carrots (98.36%) and by Ignaczak et al. [17] (95.76%). Storage of carrot snacks in edible films resulted in a statistically significant reduction in dry matter content, although samples packaged in films containing 12% gelatin showed a smaller decrease compared to those coated with 8% gelatin. Prolonging the storage time from 3 to 6 months caused an additional decrease in dry matter content, thereby increasing the differences between stored samples and the control sample and confirming the negative effect of extended storage on product stability. Alam et al. [19] also reported a decrease in dry matter content from 97% to 92–95% after 6 months of storage of dried carrot pomace–chickpea rice-based snacks packaged in plastic materials. In the present study, the use of biodegradable gelatin-based packaging, which exhibits lower barrier properties than conventional plastics, resulted in more pronounced dry matter losses during storage. The obtained results relating to dry matter changes during storage confirm the relationships obtained for water activity (Table 1 and Table 2). Nevertheless, storage at 20 °C for 3 months proved to be the most favorable condition, as confirmed by the combined analysis of water activity (lower value) and dry matter content (higher value) (Table 1 and Table 2).

Color plays an important role as a quality indicator during food processing, and can also indicate chemical and biochemical changes in the product. It is also a crucial parameter in the sensory evaluation of a product, influencing consumer preferences, acceptability, and, ultimately, food choice. Therefore, it is crucial to preserve the original color of raw materials as much as possible during food processing [20]. In the case of dried carrot snacks, color is particularly important because it indicates the degree of drying and the quality of the raw material [14]. The color also indicates the degree of the degradation of carotenoids, which are very sensitive to heat, oxygen and lipoxygenase [20,21]. Šeregelj et al. [22] confirmed the initial delay in carotene degradation in freeze-dried samples. An increase in the color saturation index after storing freeze-dried snacks for 3 months confirms the high quality of the samples (the color is attractive for the consumer) and carotenoid behavior. Extending the storage time from 3 to 6 months decreased the tested parameter. The unfavorable color change during longer carrot storage may result from the susceptibility of carotenoids to oxygen. In freeze-dried samples, which have relatively high porosity, atmospheric oxygen can easily penetrate the sample and adversely affect even the interior of the tested material [21]. This may explain the color changes in freeze-dried carrot snacks stored for 6 months. Color changes may also be related to increased water activity, which dilutes the raw material’s pigment, as reported by Silva-Espinoza et al. [23]. They studied freeze-dried snacks, which allowed us to conclude that color changes for different freeze-dried products may differ due to the composition of these products. Color changes may also be related to non-enzymatic browning, and antioxidant content/activity losses, even if the food is stored under glassy state conditions, should be shelf-stable [24].

Texture, or the set of physical properties perceptible by touch, is one of the most important and multidimensional aspects of food product quality. Texture influences the eating experience, and its perception is closely linked to the chemical composition and structure of the product [17]. It is defined as a set of physical properties related to product deformation and fragmentation, i.e., changes induced by applied force. Depending on the product type, texture can be described using various parameters, and a well-defined product texture is crucial for its consumer acceptance [25]. In the case of samples stored in edible film with a gelatin concentration of 8 and 12%, similar changes in mechanical properties were noted. Initial hardness increases after the storage of freeze-dried samples for 3 months was probably due to moisture absorption from the environment, causing structural collapse and the loss of the original porous, crisp texture [26] confirmed in the structure analysis. During the preparation of samples for the determination of the mechanical properties, it was noted that, in the case of the samples stored for 3 months, changes in the elasticity of the edible film itself occurred, which, however, effectively protected the freeze-dried snacks from changes in texture. Extending the storage time to 6 months for both temperatures resulted in a noticeable weakening of the freeze-dried product’s structure. Compression curves illustrate the effect of time and storage temperature on the reduction in sample hardness. The course of the compression curves for freeze-dried carrot snacks after storage correlates with water activity results. Samples with higher water activity had a smooth course, which was associated with changes in structure. Rowicka et al. [27] reported on the relationship between sample hardness and water activity. They noted a correlation between increasing water activity and decreasing sample hardness. Water plays a key role in shaping the hardness and structure of food products, acting as a plasticizer that increases the elasticity and reduces the hardness of materials, which is particularly important in the context of food. Depending on its content and activity, water can affect the mechanical properties of products, as emphasized in studies conducted by Sundaram and Durance [28], Moraga et al. [29], and Nowacka et al. [30]. Water is not only an essential component of food, but also a key factor influencing its hardness and structure. Understanding the role of water as a plasticizer allows for the better design and optimization of food products, which is important for their quality and consumer acceptance.

Analysis of the porosity results showed that storing freeze-dried snacks in edible films with 8 and 12% gelatin concentrations allowed for the samples to maintain a high level of porosity, in the range of 94–95%, despite changes in the structure and mechanical strength of the freeze-dried snacks. The obtained porosity values were similar to those typically reported for freeze-dried snacks without storage. Ciurzyńska et al. [31] studied freeze-dried vegetable snacks and reported porosity values ranging from 91.56 to 91.82%. The high porosity of freeze-dried samples makes them sensitive to the water vapor present in the atmosphere, which penetrates the sample, directly affecting the mechanical properties of the freeze-dried material [27], which was confirmed in the presented research. High porosity was also discussed negatively by Valentina et al. [32], who noted that high porosity increases the hygroscopicity of samples, which adversely affects the stability of stored dried material, but the high porosity of the samples stored in the gelatin films indicates that the edible packaging fulfilled a protective function.

Telis and Martínez-Navarrete [33] indicated that the freeze-dried materials, which are highly porous, are susceptible to structure changes with collapse. When a freeze-dried matrix reaches a critical temperature, which is related to glass transition temperature, a sequence of deleterious events is observed. Amorphous food materials may reach their glass transition temperature by increasing temperature and/or water content, and, above the transition temperature, various time-dependent structural transformations may occur. Changes in the structure of freeze-dried snacks observed during storage were caused by increased water activity. Water has a plasticizing effect on the products, causing the structure to begin to close [27]. This was also confirmed by changes in the mechanical properties of the dried products (Figure 3 and Figure 4). The product structure formed during processing depends on the type of raw material, its form, and the selected processing method. Freeze-dried samples are generally characterized by a delicate and porous structure, with open pores [18]. As Nowak and Jakubczyk [26] and Karwacka et al. [34] have pointed out, this set of characteristics is crucial when evaluating the attractiveness of a product. An important aspect, mentioned by Nowak and Jakubczyk [26], in shaping this characteristic is the freeze-drying process itself. In this case, the process was performed correctly, as indicated by the typical results for freeze-dried products obtained in each analysis. The increased degree of pore closure in samples stored for 6 months correlates with increased water activity, reduced dry matter content, and reduced mechanical strength, indicating that the most advantageous period for storing freeze-dried samples wrapped in edible film is 3 months.

## 4. Materials and Methods

### 4.1. Sample Preparation

The research material consisted of freeze-dried carrot snacks, whose preparation was described by Ciurzyńska et al. [13].

### 4.2. Technological Methods

#### 4.2.1. Freeze-Drying

The frozen samples were placed uniformly on the shelves of the Gamma 1-16 LSC CHRIST freeze-dryer chamber (Martin Christ GmbH, Ostrode am Harz, Germany) and subjected to freeze-drying for 24 h at a pressure of 63 Pa and a shelf temperature of 30 °C.

#### 4.2.2. Preparation of Edible Films

**Figure 8:**
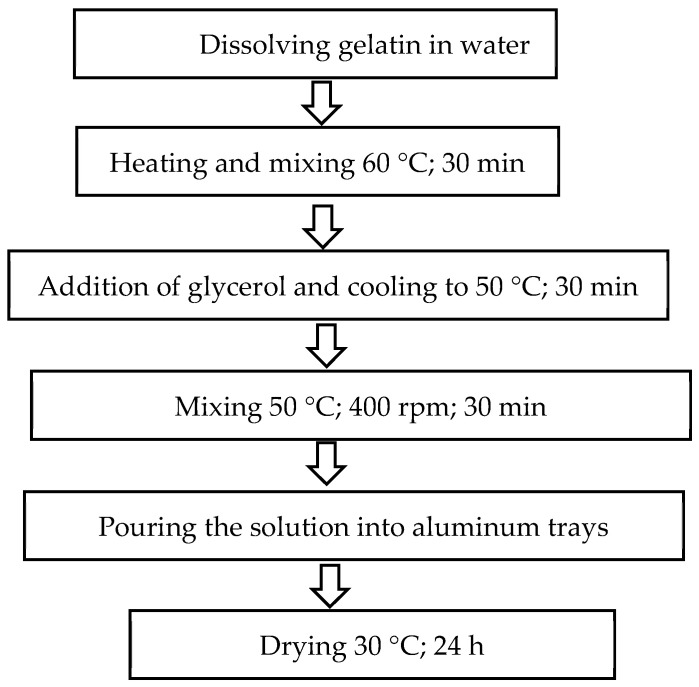
Technological diagram for obtaining edible films. Own study based on Ciurzyńska et al. [35].

Edible films were prepared based on the recipe described by Ciurzyńska et al. [35] (Table 3, Figure 8) using pork gelatin (Gelita AG, Eberbach, Germany) in two gelatin concentration variants (8% and 12%). The measured gelatin was poured into beakers, which were filled with water to a final volume of 1000 mL. The solutions were heated to 60 °C and maintained at this temperature for 30 min.

Then, glycerol (Avantor Performance Materials Poland S.A., Gliwice, Poland) was added in an amount equal to 50% of the gelatin concentration, and the mixture was cooled to 50 °C, at which it was maintained for 30 min. The solutions were mixed throughout the process using RTC basic magnetic stirrers (IKA Poland Sp. z o.o., Warsaw, Poland) at a rotation speed of 400 rpm.

The prepared solutions were poured onto aluminum trays and dried in a SUP 65 W/G dryer (WAMED, Warsaw, Poland) at 30 °C for 24 h.

#### 4.2.3. Coating of Samples

**Figure 9:**
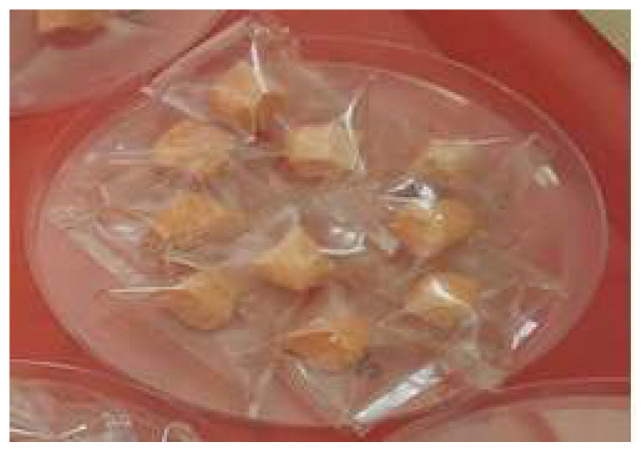
Samples prepared for storage.

The samples were wrapped in prepared edible films, the edges of which were welded using an FG.400HC sealer (SPRINTIS Schenk GmbH & Co. KG, Würzburg, Germany). The freeze-dried samples prepared in this way were placed in incubators at a refrigeration temperature of 4 °C and a room temperature of 20 °C for 3 and 6 months, respectively (Table 4, Figure 9). The storage samples were compared to the control sample which was freeze-dried carrot snack without storage and without any package to show changes in product quality after storage.

### 4.3. Analytical Methods

#### 4.3.1. Water Activity Measurement

Water activity was measured using a HygroLab C1 Rotronic meter (Bassersdorf, Switzerland) according to the manufacturer’s instructions. The measurement was performed in 4 repetitions for each sample.

#### 4.3.2. Dry Matter Content Measurement

The dry matter content was determined by the gravimetric method. The samples were crushed in a mortar and placed in weighing vessels of known mass. The prepared vessels were placed in a WAMED SUP 65 W/G dryer (WAMED, Warsaw, Poland) and dried for 24 h at 60 °C. After this time, the vessels were placed in a desiccator until they cooled down completely and weighed again. Each sample was tested in 3 replicates.

(1) $$d.m.=\left[\frac{\left(m_{3}-m_{1}\right)}{\left(m_{2}-m_{1}\right)}\right]\;\times \;100\%$$

where

d.m.—dry matter content [%];

*m*_1_—mass of empty vessel [g];

*m*_2_—mass of vessel with sample before drying [g];

*m*_3_—mass of vessel with sample after drying [g].

#### 4.3.3. Color Measurement

The color measurement was performed using a ChromaMeter CR-300 colorimeter by Konica Minolta (Yokohama, Japan) in the L*a*b* system. The measurement was performed in 5 repetitions for each sample according to the manufacturer’s instructions. Chroma was calculated according to the following equation:

(2) $$C^{*}=\sqrt{a^{2}+b^{2}}$$

where

C*—chroma index [-];

a*—red/green color coefficient [-];

b*—yellow/blue color coefficient [-].

#### 4.3.4. Mechanical Properties Measurement

The mechanical properties were measured using the TA-XT2i STABLE MICRO SYSTEMS texturometer (TA.HD plus Texture Analyser, Stable Micro Systems, Surrey, UK), an advanced device for texture analysis. The samples were tested using a 10 mm diameter hardness measuring head, at a head feed speed of 1 mm/s. The snacks were placed on the texturometer table directly under the probe, which was lowered until the deformation reached 50% of the sample height. The test was carried out at room temperature (20 °C). During the test, the results were recorded using the Texture Expert software (version 1.22). Three repetitions were performed for each sample.

#### 4.3.5. Structure Measurement

The samples were prepared by cutting a piece of the freeze-dried material with a razor blade, and then gold was sputter-coated onto the sample surface under vacuum using a Cressington sputter coater (Cressington Scientific Instruments, Tokyo, Japan) to enable imaging of the poorly electrically conductive material. Images of the samples were obtained using a TM3000 scanning electron microscope (Hitachi, Tokyo, Japan) at a magnification of 50×. All observations were conducted according to the manufacturer’s instructions.

#### 4.3.6. Porosity Measurement

The apparent density of the samples was determined using a helium pycnometer (Quantachrome Corporation, Boynton Beach, FL, USA) according to the manufacturer’s instructions. Samples of known mass but unknown volume were placed in a pycnometer chamber of known volume.

The chamber was filled with helium, which penetrated all accessible pores and void spaces within the samples. The device recorded the pressure values, and the results were processed using Pycnometer software (version 2.7). The obtained data were then used to calculate the porosity of the samples [36].

(3) $$\varepsilon =\left(1-\frac{\rho_{d}\;}{\rho_{s}}\right)\;\times \;100\%$$

where

ε—porosity [%];

*ρ_d_*—real density [kg/m^3^];

*ρ_s_*—apparent density [kg/m^3^].

#### 4.3.7. Statistical Analysis

The obtained results were subjected to three-factor analysis of variance (ANOVA) using Statgraphics software (Statgraphics Technologies, Inc., The Plains, VA, USA) with the use of Fisher’s least significant difference (LSD) procedure.

## 5. Conclusions

Analysis of the effect of storage time and temperature on the properties of freeze-dried vegetable snacks packaged in edible gelatin-based films with gelatin concentrations of 8% and 12% demonstrated that edible packaging represents a viable alternative to conventional plastic packaging. Storage of freeze-dried snacks in gelatin-based films at 20 °C proved to be the most advantageous condition, particularly for a storage period of 3 months, regardless of gelatin concentration. Under these conditions, the snacks exhibited significantly lower water activity and higher dry matter content compared to samples stored at 4 °C. Furthermore, evaluation of storage duration indicated that no significant changes occurred in the internal structure of the samples during the first 3 months of storage, as confirmed by mechanical property and porosity analyses. During this period, the samples maintained the porous and crispy structure characteristics of freeze-dried products, and color saturation values remained high, indicating good visual quality. When the storage time was extended to 6 months, although a high level of porosity was still observed, compressive strength decreased, partial pore closure occurred, and color saturation declined, primarily as a result of carotenoid degradation.

Consequently, these gelatin-based films are suitable for short-term storage (up to 3 months) at room temperature, but require secondary moisture protection for longer durations.

## Figures and Tables

**Table 1:** The effect of temperature and storage time on water activity and dry matter content of freeze-dried vegetable snacks packed in edible film based on 8% pork gelatin.

Sample Symbol	Water Activity (-)	Dry Matter Content (%)
Control	0.12 ± 0.003 ^a^	97.30 ± 0.400 ^E^
8%_4°C_3m	0.60 ± 0.005 ^d^	77.61 ± 1.060 ^B^
8%_20°C_3m	0.28 ± 0.003 ^b^	87.03 ± 2.090 ^D^
8%_4°C_6m	0.60 ± 0.005 ^d^	73.61 ± 1.960 ^A^
8%_20°C_6m	0.49 ± 0.009 ^c^	82.21 ± 1.200 ^C^

Mean values ± standard deviations. The same letter beside the values (^a–d, A–E^) indicates no statistically significant differences (*p* < 0.05). Designation of samples—Table 4.

**Table 2:** The effect of temperature and storage time on water activity and dry matter content of freeze-dried vegetable snacks packed in edible film based on 12% pork gelatin.

Sample Symbol	Water Activity (-)	Dry Matter Content (%)
Control	0.12 ± 0.003 ^a^	97.3 ± 0.400 ^D^
12%_4°C_3m	0.52 ± 0.009 ^d^	82.86 ± 4.070 ^B^
12%_20°C_3m	0.27 ± 0.004 ^b^	86.67 ± 000 ^C^
12%_4°C_6m	0.60 ± 0.008 ^e^	78.5 ± 1.530 ^A^
12%_20°C_6m	0.49 ± 0.005 ^c^	83.36 ± 1.610 ^BC^

Mean values ± standard deviations. The same letter beside the values (^a–e, A–D^) indicates no statistically significant differences (*p* < 0.05). Designation of samples—Table 4.

**Table 3:** Composition of pork gelatin film.

Composition of Film	Edible Film (8%)	Edible Film (12%)
Water	88%	82%
Gelatin	8%	12%
Glycerol	4%	6%

**Table 4:** Sample codes and explanation.

Sample Codes	Code Explanation
Control	immediately after freeze-drying, without edible film, not stored
8%_4°C_3m	packed in edible film with 8% gelatin concentration, storage temperature 4 °C, time 3 months
12%_4°C_3m	packed in edible film with 12% gelatin concentration, storage temperature 4 °C, time 3 months
8%_20°C_3m	packed in edible film with 8% gelatin concentration, storage temperature 20 °C, time 3 months
12%_20°C_3m	packed in edible film with 12% gelatin concentration, storage temperature 20 °C, time 3 months
8%_4°C_6m	packed in edible film with 8% gelatin concentration, storage temperature 4 °C, time 6 months
12%_4°C_6m	wrapped in edible film with 12% gelatin, storage temperature 4 °C, time 6 months
8%_20°C_6m	wrapped in edible film with 8% gelatin, storage temperature 20 °C, time 6 months
12%_20°C_6m	wrapped in edible film with 12% gelatin, storage temperature 20 °C, time 6 months

## Data Availability

Data will be available upon reasonable request.
